# Dysregulation of protein trafficking in neurodegeneration

**DOI:** 10.1186/1750-1326-9-31

**Published:** 2014-08-25

**Authors:** Xin Wang, Timothy Huang, Guojun Bu, Huaxi Xu

**Affiliations:** 1 Fujian Provincial Key Laboratory of Neurodegenerative Disease and Aging Research, Institute of Neuroscience, College of Medicine, Xiamen University, Xiamen, Fujian 361102, China; 2 Degenerative Disease Research Program, Sanford-Burnham Medical Research Institute, La Jolla, California 92037, USA

**Keywords:** β-amyloid precursor protein, β-secretase, γ-secretase, Sorting nexin, The retromer complex, Ras-related GTP-binding protein, Alzheimer’s disease, Down syndrome, Parkinson’s disease, Endocytic trafficking

## Abstract

Intracellular protein trafficking plays an important role in neuronal function and survival. Protein misfolding is a common theme found in many neurodegenerative diseases, and intracellular trafficking machinery contributes to the pathological accumulation and clearance of misfolded proteins. Although neurodegenerative diseases exhibit distinct pathological features, abnormal endocytic trafficking is apparent in several neurodegenerative diseases, such as Alzheimer’s disease (AD), Down syndrome (DS) and Parkinson’s disease (PD). In this review, we will focus on protein sorting defects in three major neurodegenerative diseases, including AD, DS and PD. An important pathological feature of AD is the presence of extracellular senile plaques in the brain. Senile plaques are composed of β-amyloid (Aβ) peptide aggregates. Multiple lines of evidence demonstrate that over-production/aggregation of Aβ in the brain is a primary cause of AD and attenuation of Aβ generation has become a topic of extreme interest in AD research. Aβ is generated from β-amyloid precursor protein (APP) through sequential cleavage by β-secretase and the γ-secretase complex. Alternatively, APP can be cleaved by α-secretase within the Aβ domain to release soluble APPα which precludes Aβ generation. DS patients display a strikingly similar pathology to AD patients, including the generation of neuronal amyloid plaques. Moreover, all DS patients develop an AD-like neuropathology by their 40 s. Therefore, understanding the metabolism/processing of APP and how these underlying mechanisms may be pathologically compromised is crucial for future AD and DS therapeutic strategies. Evidence accumulated thus far reveals that synaptic vesicle regulation, endocytic trafficking, and lysosome-mediated autophagy are involved in increased susceptibility to PD. Here we review current knowledge of endosomal trafficking regulation in AD, DS and PD.

## Background

Endocytic dysregulation is apparent in many neurodegenerative diseases, including Alzheimer’s disease (AD), Parkinson’s disease (PD) and Down syndrome (DS) as key examples. AD is the most common form of age-dependent neurodegeneration, affecting about 10% of the population over the age of 65 and about 50% of the population over the age of 85. Only a small subset (<10%) of AD cases is caused by inherited autosomal dominant gene mutation, and most of these familial AD mutations are found in genes encoding β-amyloid precursor protein (APP) and presenilins (PS1 and PS2) [1-4]. Accumulation of two AD hallmarks has been found in the hippocampus and cortex of AD brain, including extracellular neuritic plaques and intracellular neurofibrillary tangles (NFTs). NFTs comprise hyperphosphorylated filaments of the microtubule-associated protein tau [5]. Neuritic plaques are composed of β-amyloid (Aβ) generated through sequential proteolytic cleavage of the β-amyloid precursor protein (APP) by β- and γ-secretases [6]. APP can also be cleaved by α-secretase which cuts within the Aβ domain to preclude Aβ generation. α-secretase processing generates a secreted form of APP with neuroprotective properties. Accumulated evidence support that subcellular localization and trafficking of APP and its proteolytic secretases is critical for Aβ production. BACE1-mediated APP cleavage constitutes the rate-limiting step in Aβ generation [7]. It has been reported that BACE1 is up-regulated in human AD brain [8] and altered intracellular trafficking of BACE1 is involved in AD pathology [9-12]. A previous study suggested a mechanism for BACE1 elevation in AD where BACE1 is normally transported to lysosomes by GGA3, whereby caspase-mediated GGA3 cleavage prevents BACE1 degradation [13]. BACE1 is primarily localized in the *trans*-Golgi network (TGN) and endosomes [14], which are major cellular sites for β-secretase activity with an optimal pH value [15]. Furthermore, BACE1 is rapidly internalized from the cell surface [16] and transported to early endosomes where internalized BACE1 can be recycled by the retromer complex [17-19]. Deficiency in endocytic and recycling components will result in abnormal BACE1 trafficking and β-secretase activity. Low-density lipoprotein receptor-related proteins 1 (LRP1) is a type-I transmembrane glycoprotein. It has been demonstrated that LRP1 can affect APP trafficking and processing through APP binding interactions with LRP1 extracellular and intracellular domains [20-22]. Further understanding of AD-related protein trafficking and regulation would provide new approaches for AD therapy.

Down syndrome (DS) is a congenital disorder that affects multiple organs and causes developmental delay and mental retardation [23,24]. Patients with DS have an extra copy of chromosome 21, leading to an over-production of gene products and non-coding RNAs encoded by this chromosome. These include APP, Dual specificity tyrosine-phosphorylation-regulated kinase 1A (DYRK1A), runt-related transcription factor 1 (RUNX1), and other chromosome 21-encoded components [23]. Over-production/accumulation of Aβ (a proteolytic product of APP) in the brain is considered as a key factor in AD pathogenesis. Similarly, all DS patients develop an AD-like neuropathology by the age of 40, including extracellular amyloid plaques, intracellular neurofibrillary tangles and synaptic dysfunction. Endocytic dysfunction is an early pathological event in Alzheimer’s disease (AD) and Down’s syndrome (DS). In previous studies, investigators found that both primary fibroblasts from DS individuals and neurons from DS mouse models exhibit abnormal endocytic and lysosomal trafficking [25,26]. Although several chromosome 21-encoded products such as APP and synaptojanin 1 (SYNJ1) are thought to contribute to these defects [26,27], the detailed molecular mechanisms by which trisomy 21 results in dysfunction of the endocytic trafficking remains largely unclear.

Parkinson’s disease (PD), the second most common neurodegenerative disease, affects more than 4 million people worldwide. PD is characterized by a series of motor symptoms, including akinesia, rigidity, postural disturbance and tremor [28]. Motor deficits associated with PD result from the loss of dopaminergic neurons in the substantia nigra subregion of the midbrain. Inherited genetic mutation and environmental toxins have both been reported to be causal to dopaminergic neuronal death. Although most PD patients arise from sporadic cases, less than 10% of familial cases are caused by single monogenic mutations [29]. Several causative mutations have been identified in rare inherited familial PD [30,31]. For example, autosomal dominantly inherited mutations in α-synuclein (α-syn), including missense mutations and triplication of the α-synuclein locus, are found in familial forms of inherited PD. Autosomal dominantly inherited mutations in leucine-rich repeat kinase-2 (LRRK2) gene are associated with an increased risk of PD. LRRK2 is a member of the leucine-rich repeat kinase family with GTPase and kinase activities. How these components contribute to PD neuropathology in a protein trafficking context is described below.

### Endocytic sorting in neurodegenerative diseases

Full-length APP is a type I transmembrane protein synthesized in the endoplasmic reticulum (ER) and subsequently transported to TGN [32,33]. APP can be delivered from the TGN to the cell surface where it is cleaved by α-secretase to generate a neuroprotective, non-amyloidogenic sAPPα fragment [34]. Several ADAM (a disintegrin and metalloproteinase) family members possess α-secretase activity and three ADAM-family α-secretases have been confirmed so far: ADAM9, ADAM10, and ADAM17. APP can also be re-internalized via an endosomal/lysosomal degradation pathway [35]. The neurotoxic Aβ peptide is generated through sequential cleavage by β-secretase (BACE1) and the PS1/γ-secretase complex in the ER, Golgi/TGN [33] as well as the endosomal/lysosomal system [36,37]. As the subcellular distribution of APP plays a key role in Aβ generation, delineation of the mechanisms involved in APP trafficking is thus relevant and crucial to understanding the pathogenesis of AD.

Several PD-linked mutations have been found to be associated with LRRK2 and α-synuclein genes, and both LRRK2 and α-synuclein have been reported to play important roles in protein sorting in neurons. For instance, PD-associated LRRK2 mutations are implicated in protein degradation defects in lysosomes, suggesting that LRRK2 may affect delivery of cytosolic proteins and protein aggregates to the lysosome [38-40]. In addition, it has been reported that LRRK2 mutations may also induce Golgi fragmentation [41]. It has also been reported that α-synuclein affects dopamine release in dopaminergic neurons, and *α-Syn*^*-/-*^ mice display altered dopamine release [42]. Furthermore, expression of α-syn in yeast and mammalian cells blocks protein transport from the endoplasmic reticulum (ER) to Golgi apparatus [43,44]. Taken together, these findings suggest that defective protein transport in intracellular compartments plays a role in PD.

### Trafficking components and neurodegeneration

#### The retromer complex, SorLA and GGA1

The Retromer complex is composed of the vacuolar protein sorting (VPS) trimer core sub-complex (VPS26, VPS29, VPS35) and a membrane-associated sorting nexin (SNX) dimer (SNX1, SNX2, SNX5, SNX6) [45]. The retromer complex has been shown to be important in regulating transmembrane receptor recycling from endosomes to TGN. The SNX dimer is required for the recruitment of the retromer complex to the endosomal membrane, and the VPS35 subunit is presumed to be the core cargo-binding component with binds a variety of cargo proteins [46], including CI-M6PR [47], wntless [48-50] and sortilin [51].

Expression of two components of the retromer complex, VPS26 and VPS35, is reduced in the brains of individuals with AD [52]. Cell culture studies showed that over-expression of VPS35 down-regulated Aβ generation, and VPS35 depletion using small interfering RNAs up-regulated Aβ peptide levels [52]. Further studies revealed that retromer deficiency promotes Aβ generation and exacerbates neurodegeneration by modulating BACE1 activity in Vps26 and Vps35 knockout mouse models [18,19]. Moreover, recent research indicates that a chemical chaperone can decrease APP processing and Aβ generation through stabilizing the retromer complex and hence transporting APP away from endosomes [53].

Recently, a missense mutation in the VPS35 subunit (D620N) has been identified in multiple families with late-onset Parkinson’s disease (PD) [54-56]. Further mechanistic studies revealed that the VPS35 D620N mutation may redistribute retromer-positive endosomes to a perinuclear subcellular localization. In support of this notion, enlarged endosomes have been found in the fibroblasts isolated from a PD patient with the D620N mutation [57]. Moreover, over-expression of a VPS35 D620N mutant construct disrupts the trafficking of cathepsin D [57], the main lysosomal enzyme for degrading α-synuclein [58]. This may suggest that the late-onset PD linked VPS35 D620N mutation leads to endosomal alterations and trafficking defects in patient fibroblasts. The generation of a Vps35 D620N knock-in mouse model and patient-derived induced pluripotent stem (iPS) cell models may provide new strategies to better understand the relevance and mode of action of the D620N VPS35 PD allele.

The Sortilin-related receptor with A-type repeats SorLA (also known as SORL1, LR11) is a type I membrane protein. Reduced SorLA expression has been found in the brains of AD patients [59] and some inherited variants of the *SorLA* gene have been found to associate with late-onset AD [60]. Although the function of SorLA in AD pathology is unclear yet, it has been reported that SorLA is involved in APP processing. SorLA modulates recycling of APP and prevents amyloidogenic processing of APP as down-regulation of SorLA increases sorting of APP into Aβ-generating compartments [60], while SorLA-deficient mice show increased levels of Aβ [61]. Further, SorLA may regulate APP sorting and processing through interactions with the VPS26 subunit of the retromer complex [62]; VPS26 binding to a cytosolic SORLA tail motif may be important for SorLA-mediated APP retention at the Golgi. Disruption of these interactions results in APP sorting to non-Golgi compartments and increased amyloidogenic APP processing [62]. Recently, the Aβ peptide has been shown to interact directly with the SorLA Vps10 domain, which then directs the Aβ peptide to the lysosome for consequent clearance and degradation [63]. Together, these results indicated that SorLA is an important trafficking component of APP, and may have dual functions in retaining APP at the Golgi, regulating amyloidogenic APP processing and directing the Aβ to lysosomal compartments for subsequent degradation.

ADP-ribosylation factor-binding proteins (GGAs) are a family of Golgi-localized monomeric clathrin adaptor proteins that are involved in the transport of cargo proteins from the TGN to the endosome [64]. Mammalian GGAs (GGA1, GGA2, and GGA3) contain three domains, including a N-terminal VHS domain, an intermediary GAT (GGA and Tom1) domain and a C-terminal GAE (γ-adaptin ear) domain [64].

The GGA VHS domain can recognize a BACE1 DISLL motif located within the BACE1 cytoplasmic domain (aa 496–500) [10,65]. Previous studies indicate that phosphorylation of BACE1 is important for GGA1-mediated BACE1 endosomal trafficking; phosphorylated BACE1 can be efficiently transported from endosomes to TGN, whereas non-phosphorylated BACE1 is recycled directly from endosomes to the plasma membrane [65-67]. Over-expression of GGA1 reduces Aβ secretion, while knockdown of GGA1 increases Aβ secretion in HEK293 cells [66]. In addition, it has been shown that only GGA1 but not GGA2 and GGA3 can regulate intracellular distribution of SorLA and APP in the endocytic recycling compartments [68]. BACE1 S498A mutation enhances BACE1 targeting to SorLA-positive compartments and attenuates SorLA-mediated reduction of Aβ [68]. However, unlike GGA1, it has been found that GGA3 mediates trafficking of BACE1 to lysosomes for degradation [13,69,70]. It has been reported that ubiquitination of BACE1 at K501 is important for GGA3-mediated BACE1 trafficking to lysosomes and BACE1 stability [71]. In support of this, down-regulation of GGA3 increases BACE1 expression [13,70]. In AD brains, GGA3 level is markedly down-regulated and negatively correlates with BACE1 expression levels. Recently, it has been reported that a small GTPase ADP ribosylation factor 6 (ARF6) is important for regulating the internalization of BACE1 into early endosomes to promote BACE1-mediated APP cleavage. To facilitate this process, the BACE1 DISLL motif is required for BACE1 sorting from ARF6-positive endosomes to RAB5-positive endosomes [72].

#### Sorting nexins in APP processing and synaptic dysfunction

**Figure 1:**
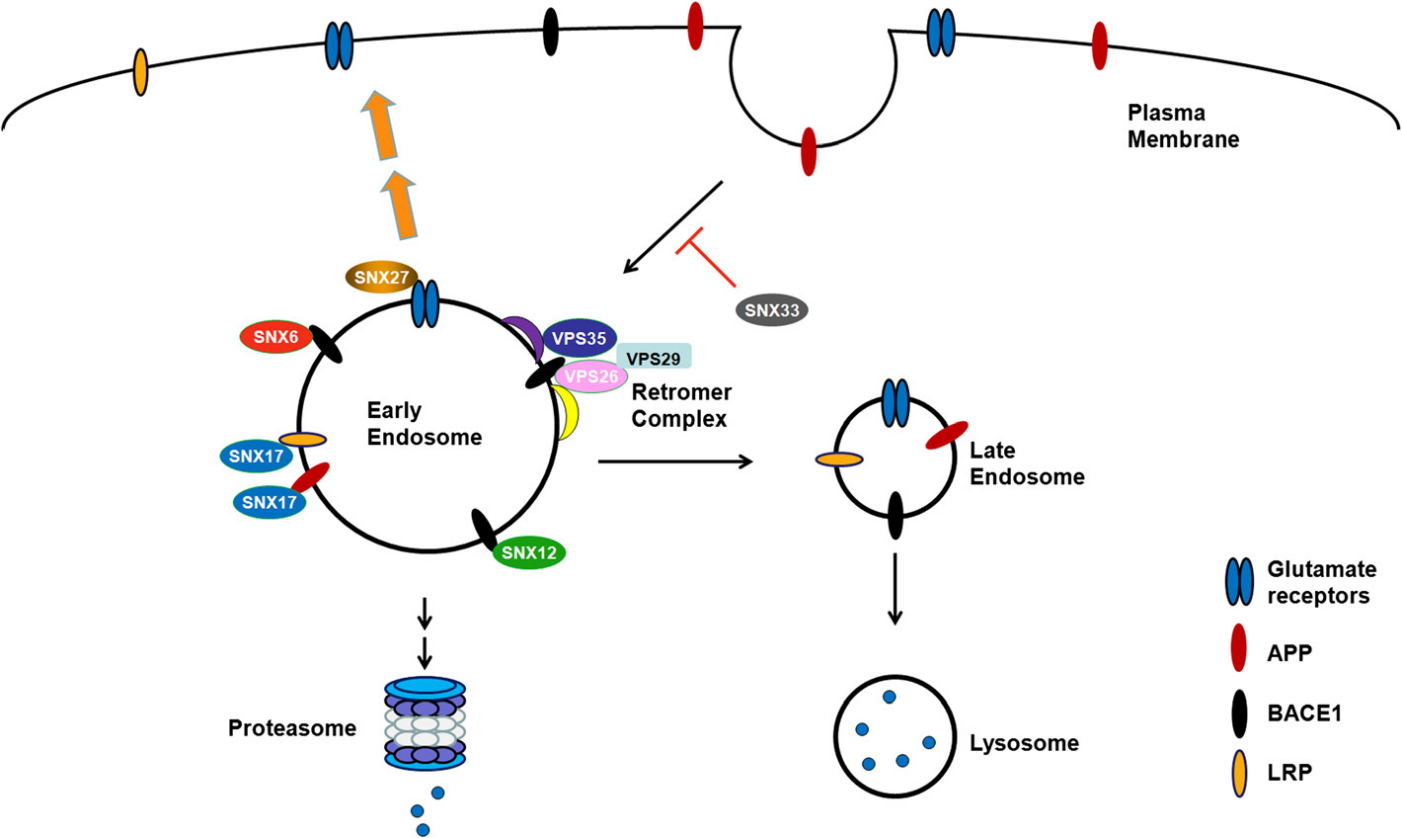
**Regulation of AD-associated proteins by sorting nexins and the retromer complex.** SNX6, SNX12, SNX17 and SNX27 regulate cell surface delivery of several AD-associated proteins, including APP, BACE1, glutamate receptors and LRP. SNX33 inhibits APP endocytosis in a dynamin-dependent manner. Over-expression of SNX33 up-regulates cell surface APP levels and increases α-secretase cleavage of APP. The retromer complex regulates APP processing and Aβ generation through modulating BACE1 trafficking and activity.

The sorting nexin family of trafficking components comprise 33 family members, each containing a signature lipid-binding PX domain [73]. At least 5 sorting nexins have been found to regulate APP cleavage or Aβ production (Figure 1). Sorting nexin 17 (SNX17) was the first identified sorting nexin in the regulation of APP trafficking and processing [74]. In early endosomes, SNX17 regulates APP endocytosis through specific binding to the YXNPXY motif in the APP cytoplasmic domain. SNX17 loss-of-function through over-expression of a dominant-negative mutant of SNX17 or siRNA knockdown of SNX17 in human glioblastoma U87 cells reduced steady-state APP levels and increased Aβ production. In addition, SNX17 can regulate cell surface delivery of LRP by promoting its recycling from early endosomes [75]. The FERM domain and the carboxyl-terminal region of SNX17 is required for LRP binding, and SNX17 binds to the cytoplasmic tail NPxY motif of LRP. Functional mutation of the NPxY motif reduced LRP recycling from endosomes but did not influence LRP endocytosis. Likewise, knockdown of SNX17 using siRNA also disrupted LRP recycling.

SNX33 was identified as a new activator of APP α-secretase cleavage [76]. Over-expression of SNX33 in cultured HEK293 and COS cells markedly increased APP α-secretase cleavage but did not affect on β-secretase cleavage. SNX33 has been found to bind the endocytic GTPase component dynamin to reduce APP endocytosis in a dynamin-dependent manner. Increased cell surface expression of APP results in enhanced α-cleavage upon SNX33 over-expression. It is anticipated that future studies will investigate SNX33 loss-of-function and its effect on APP processing.

Using a tandem affinity purification-based proteomic approach, SNX6 was identified as a BACE1-associated protein [77]. Interestingly, SNX6 is a putative component of the retromer complex. Knockdown of SNX6 increased generation of β-cleavage products of APP, including Aβ, sAPPβ and β-CTF. Furthermore, reduction of SNX6 stabilized BACE1 and promoted retrograde transport of BACE1 from the cell surface to perinuclear vesicles.

SNX12 is highly expressed in brain tissues and is mainly localized in early endosomes [78]. Over-expression of SNX12 reduced Aβ levels, soluble APPβ and APP β-carboxyl terminal fragments, but did not affect steady-state levels of APP, BACE1 or γ-secretase components [79]. Conversely, down-regulation of SNX12 by siRNA transfection reverses these effects. Modulation of SNX12 levels has little or no effect on γ-secretase activity or *in vitro* β-secretase activity. Further studies reveal that SNX12 interacts with BACE1 and down-regulation of SNX12 accelerates BACE1 endocytosis and decreases steady-state cell surface BACE1 levels. Importantly, SNX12 protein levels are markedly reduced in human brain tissue from sporadic AD patients.

SNX27 is a brain-enriched sorting nexin component, and is the only sorting nexin family member containing a PDZ domain. *Snx27* is essential for normal development and survival in mammals, as *Snx27*^*-/-*^ mice display developmental retardation phenotypes [80] and severe neuronal pathology in the hippocampus and cortex [81]. *Snx27*^*+/-*^ mice comprise a normal neuroanatomy overall, but demonstrate defects in synaptic function, learning and memory accompanied with a reduction in the ionotropic NMDA and AMPA class glutamate receptors. SNX27 interacts with these receptors through its PDZ domain [81,82], regulating their recycling to the plasma membrane. Interestingly, reduced expression of SNX27 and its upstream regulatory transcription factor CCAAT/enhancer binding protein β (C/EBPβ) has been observed in Down syndrome brains. Over-expression of the chromosome 21-encoded microRNA, miR-155 in trisomy 21 results in the attenuation of C/EBPβ expression, thereby reducing SNX27 levels, resulting in concomitant synaptic dysfunction. Restoration of SNX27 in the hippocampus of Ts65Dn Down syndrome mouse models rescues synaptic and cognitive deficits. In addition to its role in synaptic function, we also found that SNX27 deficiency enhances PS1/γ-secretase complex formation and increases γ-secretase abundance and activity to elevate Aβ production both *in vitro* and *in vivo* (unpublished data).

#### Mint family and Ras-related GTP-binding (Rab) proteins

The Mint (Munc18 interacting protein, also known as X11) adaptor protein family includes three members: neuron specific Mint1 and Mint2, and the ubiquitously expressed Mint3 [83,84]. All three Mint proteins consist of a phosphotyrosine binding (PTB) domain and two tandem PDZ (postsynaptic density-95/discs large/zona occludens-1) domains. Evidence so far indicates that the Mint family is involved in neuronal protein transport and synaptic function [85-87]. Mint proteins can interact with the APP C-terminus (YENPTY motif) through the PTB domain binding. APP interaction with mint proteins has been found to influence APP trafficking/processing and Aβ generation *in vitro* and *in vivo*[87-89]. In addition, Mint1 and Mint2 have been reported to bind to presenilin1 through their PDZ domains [90,91] and Mint proteins potentially inhibit γ-secretase-mediated APP cleavage through direct interactions. However, a detailed mechanism how this occurs is yet lacking.

Several Rab GTPase components have been found to regulate APP processing and Aβ production. Rab1B plays a key role in the transport of APP or APP β-CTF from the endoplasmic reticulum to the Golgi; expression of a dominant-negative mutant of the Rab1B almost completely eliminates Aβ generation [92,93]. Rab6 is involved in intra-Golgi vesicular trafficking and a Rab6 N126I dominant negative mutant has been found to enhance amyloidogenic APP processing [94]. It has been recently shown that EH domain-containing proteins (EHDs) and Rab11 facilitate BACE1 trafficking in dendrites and axons in primary neurons [95-97]. Several Rab proteins have been reported to be involved in PS1-mediated protein trafficking, such as Rab11 [98], Rab6 [99] and Rab GDP dissociation inhibitor [100].

Enlarged early endosomes, increased immunoreactivity for early endosome markers (rab5, EEA1 and rabaptin5), and the recycling endosome marker rab4 have been observed in the neurons of a Ts65Dn DS mouse model [26]. In addition, increased endocytic uptake, fusion, and recycling have also been found in DS human fibroblasts. Moreover, DS fibroblasts show an increased number of enlarged endosomal vesicles enriched with the late endosome marker rab7 [25]. These changes strikingly resemble neurons from both AD and DS brains. Interestingly, over-expression of a rab5 mutant that inhibits endocytic uptake reversed endosomal abnormalities in DS fibroblasts.

Recently, it has been reported that deficiency of the PARK16 locus gene RAB7L1 is involved in PD neuropathology. RAB7L1 over-expression rescues LRRK2 mutation-induced phenotypes in a drosophila PD model [101]. Expression of the VPS35 retromer component could rescue the endosomal-lysosomal sorting defects caused by mutant LRRK2 or RAB7L1 *in vitro* and *in vivo*[101]. Together, these results indicate that various trafficking components such as RAB7L1 and VPS35 can exert protective effects on pathological PD components such as LRKK2.

#### The roles of PS1 in autophagy

Autophagy is a catabolic pathway triggered by starvation and involves degradation of cellular components through the lysosome. Autophagy is involved in eliminating damaged organelles and misfolded protein aggregates, and removes unnecessary cellular components to liberate available nutrients during starvation. As an essential process in neuronal survival, dysfunction in the autophagic response has been found to contribute to neurodegeneration. It has been reported that the AD-related protein PS1 is required for autophagy [102,103], and loss of PS1 can result in impaired proteolytic activation and autophagosome clearance. Familial AD-associated PS1 mutations commonly found in early-onset AD may affect lysosomal function and accelerate neurodegenerative progression [104,105]. Defective lysosomal proteolysis may trigger accumulation of toxic proteins and cause neuronal cell death in AD and other neurodegenerative diseases. However, the mechanisms underlying these processes remain controversial. Lee et al. found that deficits in the autophagy pathway may be caused by impaired PS1-dependent delivery of the v-ATPase V0a1 subunit to lysosomes, thereby attenuating autolysosome acidification and cathepsin activation [106]. Coen et al. showed that N-glycosylation may not be necessary for targeting and normal function of the V-ATPase subunit, and that defective N-glycosylation of V0a1 and lysosomal acidification may not be the cause of endo-lysosomal dysfunction in PS1/2 dKO cells. Rather, a disruption in lysosomal calcium storage and release was found to be impaired in PS1/2 dKO cells, thereby contributing to autophagic defects [107]. In addition, transcriptome analysis of PS1/2 dKO mouse brains revealed a role for presenilins in regulating lysosomal biogenesis [108]. Although how presenilins are involved in autophagic processes in neurodegeneration remains unclear at this point, new aspects of presenilins in autophagy will be surely uncovered in future studies.

## Conclusion

Although endocytic trafficking has been well-studied in the last few decades, regulation of protein trafficking in the context of neurodegenerative diseases is far from clear. For example, as a well-characterized substrate of α-, β- and γ-secretases, APP and its metabolites play a critical role in AD pathology. Cumulative evidence demonstrates that APP cleavage by different secretases may occur at distinct subcellular compartments, implicating the importance of the subcellular distribution of APP and various secretases in regulating Aβ generation. Trafficking regulation in neurodegenerative diseases is a complicated process in which a number of regulators, motor molecules and membrane proteins are involved. Despite the characterization of several common defects in protein sorting and neuropathology found in DS and AD so far, further studies are anticipated to uncover unique trafficking pathways for DS and AD. In addition, future studies are needed to determine how PD-associated gene mutations can affect membrane vesicle trafficking, and more importantly vesicular trafficking of neurotransmitters to cause dopaminergic dysfunction. This review covers some aspects of endocytic trafficking regulation in several disease-associated proteins, including APP, secretases, glutamate receptors and LRRK2. Future research is expected to strengthen our understanding of dysregulated protein trafficking in neurodegeneration and may potentially provide new prevention or treatment strategies.

## Abbreviations

Aβ: β-amyloid; AD: Alzheimer’s disease; ADAM: A Disintegrin and Metalloprotease; AICD: APP intracellular domain; APP: Amyloid beta (A4) precursor protein; ARF6: ADP ribosylation factor 6; BACE1: Beta-site APP-cleaving enzyme 1; C/EBPβ: CCAAT/enhancer-binding protein beta; CD-M6PR: Cation-dependent mannose-6-phosphate receptor; CTF: Carboxyl-terminal fragment; DS: Down syndrome; EHDs: EH domain-containing proteins; ER: Endoplasmic reticulum; GAE: γ-adaptin ear; GGA: Golgi-localised γ-adaptin ear-containing ADP ribosylation factor-binding proteins; LRRK2: Leucine-rich repeat kinase 2; LRP1: Low-density lipoprotein receptor-related proteins 1; Mint: Munc18 interacting protein; NFTs: Neurofibrillary tangles; NTF: Amino-terminal fragment; PDZ: PSD-95, Drosophila disks-large, ZO-1; PLD1: Phospholipase D1; PS1: Presenilin 1; PTB: Phosphotyrosine binding; Rab: Ras-related GTP-binding protein; SNX: Sorting nexin; SORLA: Sortilin-related receptor, (LDLR class) A repeats containing; TGN: *Trans*-Golgi network; VPS: Vacuolar protein sorting-associated protein; VHS domain: VPS-27, Hrs and STAM domain.

## Competing interests

The authors declare that they have no competing interests.

## Authors’ contributions

XW, TH and HX wrote and revised, and GB discussed and revised the manuscript. All authors read and approved the final manuscript.
